# Cutaneous Leishmaniasis in the Immunocompromised: Diagnostic and Therapeutic Insights from a Case Documented in Central Italy

**DOI:** 10.3390/idr17050125

**Published:** 2025-10-08

**Authors:** Laura Povolo, Anna Barbiero, Michele Spinicci, Nicola Petrosillo, Alessandro Bartoloni, Lorenzo Zammarchi

**Affiliations:** 1 Department of Experimental and Clinical Medicine, University of Florence, 50121 Florence, Italy; laura.povolo@unifi.it (L.P.); anna.barbiero@unifi.it (A.B.); michele.spinicci@unifi.it (M.S.); lorenzo.zammarchi@unifi.it (L.Z.); 2 Regional Referral Center for Tropical Diseases, Infectious and Tropical Diseases Unit, Careggi University Hospital, 50134 Florence, Italy; 3 Infectious and Tropical Diseases Unit, Careggi University Hospital, 50134 Florence, Italy; 4 Infection Prevention & Control/Infectious Disease Service, Fondazione Policlinico Universitario Campus Bio-Medico, 00128 Rome, Italy; n.petrosillo@policlinicocampus.it

**Keywords:** cutaneous leishmaniasis, immunocompromised, Italy, *Leishmania infantum*, cutaneous swab, PCR

## Abstract

Introduction: Cutaneous leishmaniasis (CL) poses a number of challenges when it comes to diagnosis and treatment, due to the variety of clinical presentations that mimic other conditions and hinder the choice of the most appropriate therapeutic approach, especially in the context of immunodepression. Case presentation: We present the case of a 63-year-old woman on anti-tumor necrosis factor (TNF) therapy, who underwent surgical excision for the diagnostic purposes of a chronic non-healing lesion located on her right arm. The histopathological examination revealed the presence of *Leishmania* amastigotes. CL relapsed in the following months, with new lesions appearing both close to the excision scar and at a different body site. At this point, in order to avoid another surgical intervention, cutaneous swabs for *Leishmania* Polymerase Chain Reaction (PCR) were performed on both lesions. Both samples yielded positive results, and the patient was treated with a 4-week course of miltefosine. Conclusions: These results support the use of cutaneous swabs as a highly sensitive and less invasive tool for the diagnostic workup of CL. In addition, our case prompts a reflection on the management of immunosuppressed patients with CL, with particular emphasis on the risk of reactivation or simultaneous involvement of multiple anatomical sites, thus suggesting the need for specific considerations and personalized management for this group of subjects.

## 1. Introduction

Leishmaniasis, caused by the obligated intracellular protozoa of the genus *Leishmania* spp., can present with a wide clinical spectrum that includes visceral and tegumentary forms. In Southern Europe, the only endemic species is *Leishmania infantum*, which may be responsible for visceral leishmaniasis (VL), cutaneous leishmaniasis (CL), and, less frequently, mucocutaneous leishmaniasis (ML) [1,2]. CL usually presents with indolent and chronic evolution; however, it can evolve into disfiguring and dysfunctional scars, as well as recurrent or disseminated disease in immunocompromised patients [3,4].

In Italy, *L. infantum* has historically been endemic in southern regions, the big islands (Sardinia and Sicily), the Tyrrhenian coast, and, to a lesser extent, the Adriatic coast [5]. However, an increasing spread of the diseases caused by this parasite to central and northeastern regions has recently been widely reported [5,6,7,8,9,10], likely related to environmental, epidemiological, and climate changes in these areas. In the Tuscany region, central Italy, a recent study showed an increase in the overall incidence of human leishmaniasis from 0.22 autochthonous cases per 100,000 inhabitants in 2018 to 1.81/100,000 in 2023, 27% of which were cases of CL [9].

Early diagnosis of CL is crucial to limit disease progression, mucosal involvement, and development of disfiguring scars, as well as severe disease complications in immunocompromised patients [10]. However, lack of awareness both among patients and clinicians and limited access to effective diagnostic techniques often lead to prolonged and complicated diagnostic paths before receiving appropriate diagnosis and treatment. Traditionally, CL diagnosis has been based on the microscopic or molecular demonstration of *Leishmania* spp. on biopsy samples, while a few studies have evaluated the use of non-invasive sampling methods, such as tape disks, cytology brushes, or swabs, which could represent a less invasive, easier to perform, and less time- and resource-intensive diagnostic approach for an effective diagnosis of CL [11,12,13].

With regard to treatment, the main goal is to reduce morbidity in terms of relapses, scarring, or dissemination. Since there is no single drug of choice [11,14], therapeutic decisions need to be personalized according to the type of patient, taking into consideration pre-existing conditions such as underlying immunosuppression or significant comorbidities, in addition to the characteristics, number, and site of the lesions, and lastly the causative *Leishmania* species [15]. CL poses even more challenges in the diagnosis and management of immunosuppressed patients, as its clinical presentation is strongly dependent on both parasitic and host factors, especially the host immune system. Hence, immunosuppressive drugs, especially tumor necrosis factor (TNF) inhibitors, can favor the development of clinically evident leishmaniasis as well as relapses after appropriate treatment. TNF is an inflammatory cytokine that has a crucial role in enhancing macrophage activation and granulomas formation; its blockage through immunomodulatory therapies can elicit an increased replication of the parasite and promote infection reactivation [16].

## 2. Case Description

**Figure 1:**
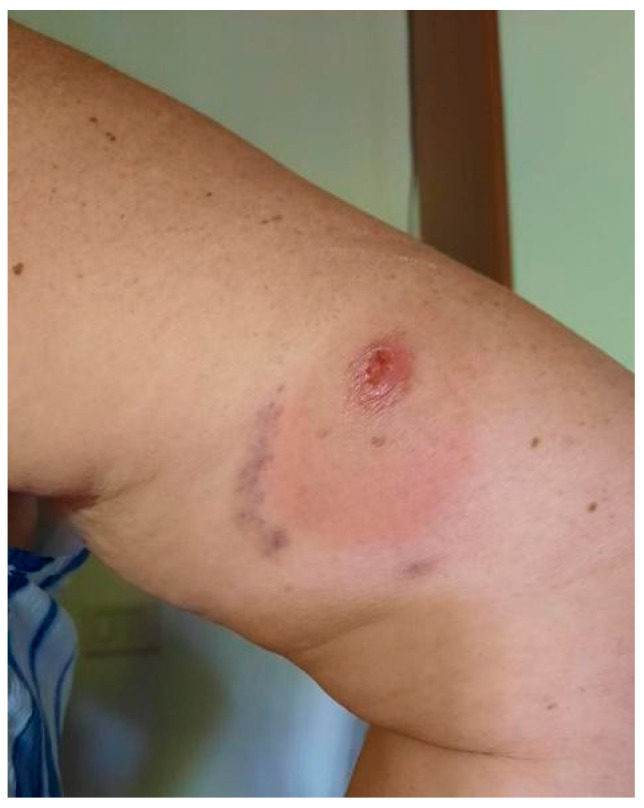
First painless ulcerated lesion appeared two years before its complete surgical excision.

In April 2025, a 63-year-old woman was referred to the Regional Referral Center for Tropical Diseases, Tropical and Infectious Diseases Unit, Careggi University Hospital (Florence, Italy), suspect of CL. Two years earlier, she had reported the appearance of a small painless ulcerated lesion on the upper part of her right arm. It progressively increased in size up to a diameter of around 2 cm (Figure 1) despite multiple courses of topical steroid and antibiotic treatments with betamethasone and gentamicin and one course of systemic antibiotic treatment with oral amoxicillin/clavulanic acid.

In her past medical history, the patient reported rheumatoid arthritis currently on treatment with adalimumab, started in 2024, after three years of treatment with methotrexate. She had no history of travel outside Europe.

In September 2024, she underwent an excisional biopsy on suspicion of basal-cell carcinoma. The histopathological examination showed the presence of a chronic, non-necrotizing granulomatous inflammatory process with multinucleated giant cells, numerous plasma cells and round intracytoplasmic CD1a reactive bodies, compatible with amastigotes, thus suggesting a diagnosis of CL.

The presence of *Leishmania* spp. was confirmed through a Polymerase Chain Reaction (PCR) and the species was identified as *L. infantum*. Serology for *L. infantum* was also performed, and it resulted negative. Information on kits and methods used for *Leishmania* species identification and serology at this time are not available, since these assays were performed in another center.

At that time, no specific treatment was administered since the lesion appeared to have completely resolved following the excisional biopsy.

**Figure 2:**
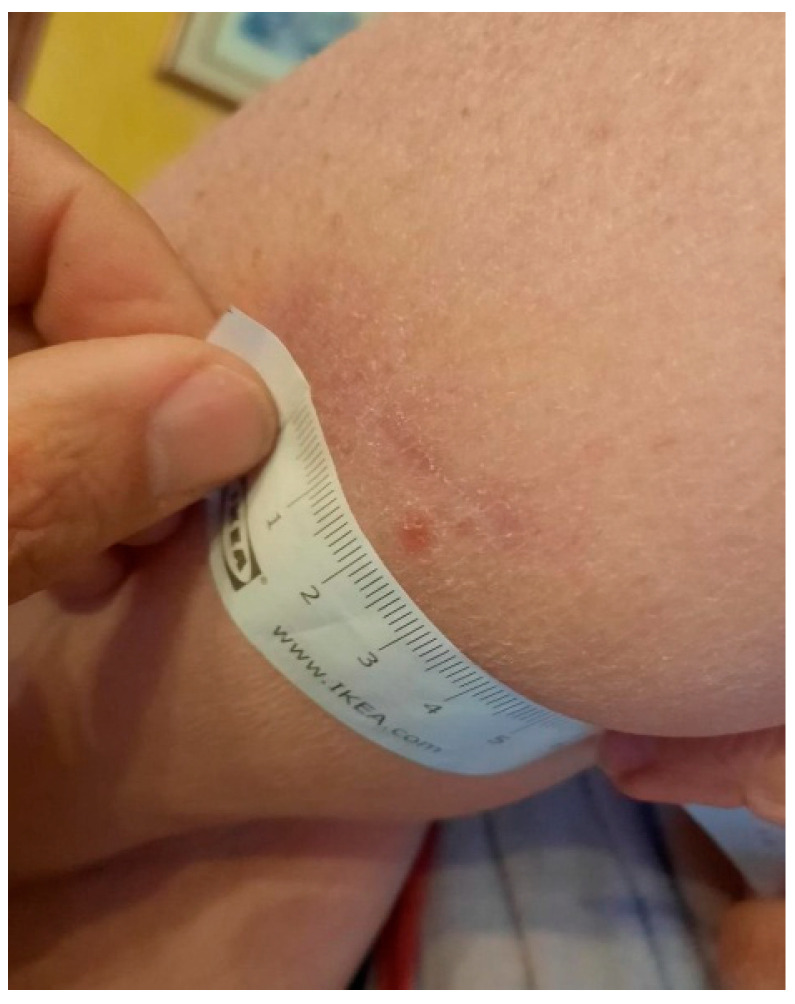
Nodular recurrent lesion appearing next to surgical scar six months after the excision.

**Figure 3:**
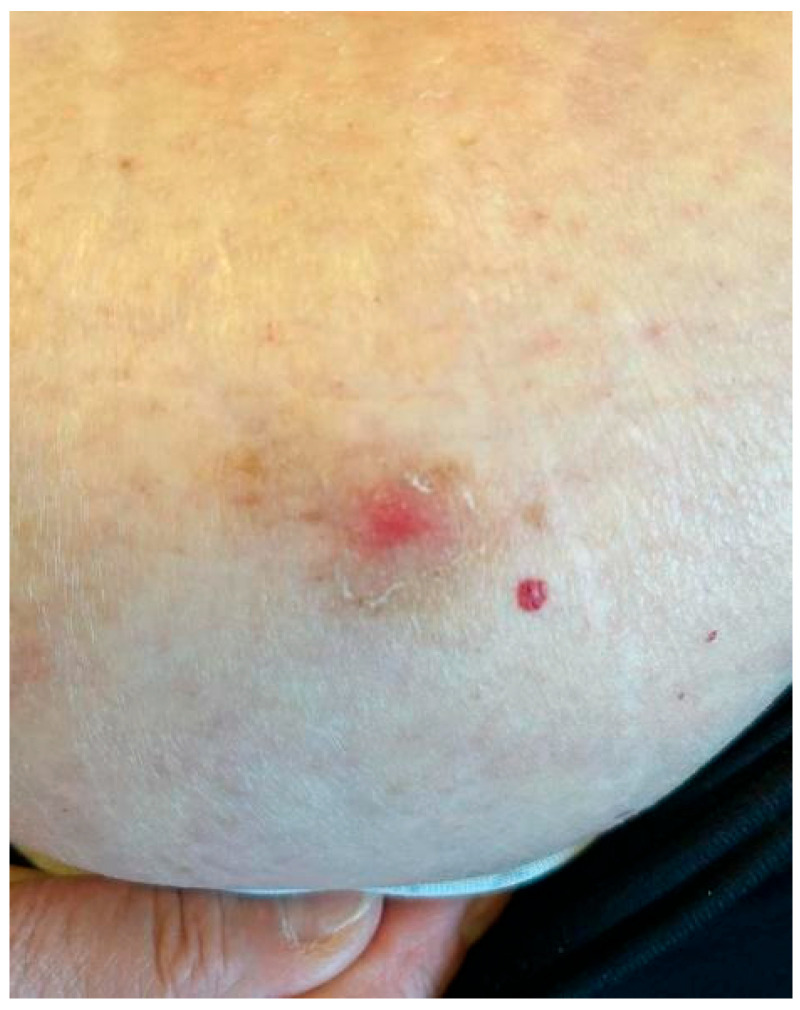
Hyperkeratotic CL recurrence in the abdominal region.

After about six months, two new suspicious lesions appeared, and the patient was referred to our center. One of the two lesions was located on the right arm, near the healed scar of the cutaneous biopsy; it appeared as a small, non-ulcerated and non-exudative nodule measuring around half a centimeter (Figure 2). The other lesion, located in the abdominal region, appeared as a hyperkeratotic crusted non-exudative papule (Figure 3). A cutaneous swab was obtained after scraping each lesion with a blazer in order to uncover the underlying skin tissues; the swab was then rubbed in a clockwise direction over the lesion surface (especially on the margins). *Leishmania* PCR was then performed on the obtained swabs, using a qualitative Real-Time PCR kit (Clonit SRL, Milan, Italy) targeting the 18S ribosomal RNA sequences of the Leishmania genome. Both swabs yielded positive results.

Considering the relapse, the patient underwent treatment with miltefosine at a dose of 50 mg every 8 h for 28 days without interruption of immunosuppressive treatment. Glucantime administration was unfeasible due to practical limitations, since it would have required weekly visits to our center, located at a significant distance from where the patient lives. At the follow-up visit two months after the end of treatment, the abdominal lesion was found to be completely healed, whereas the arm lesion appeared essentially unchanged. The patient will be re-evaluated three months after the end of treatment. In case of non-healing or failure to improve skin lesions, a second-line therapy and/or discontinuation of the immunosuppressive treatment will be considered.

## 3. Discussion

The described case shows the challenges that are often faced by clinicians and patients for the diagnosis of CL, especially in the context of immunodepression: the patient went through a long and difficult medical journey before receiving appropriate diagnosis and treatment.

On one hand, low awareness among clinicians and patients regarding the presence of the disease in the Mediterranean area often causes misdiagnoses and sometimes implicates invasive procedures due to the suspicion of malignities. This usually leads to a relevant prolongation of the time to diagnosis and the start of an adequate treatment [9]. A study recently conducted in the same study area indeed showed a median time to diagnosis of 174 days for autochthonous cases of CL [9]. On the other hand, even though non-invasive sampling methods combined with molecular analysis are gaining increasing interest worldwide [17], current guidelines indicate that direct demonstration of *Leishmania* spp. through smear microscopy, culture, PCR, or histology on skin biopsy represents the standard diagnostic tool for CL [10,11,14]. These elements often lead to the use of invasive procedures (i.e., biopsies) for the diagnosis of the disease, as in the described case, that are much more uncomfortable for the patients, as well as resource- and time-intensive for clinicians. Conversely, it has been shown that the use of PCR-based swab sampling can achieve an accuracy similar to that of biopsy samples [18] and could prevent an invasive and painful procedure [19], especially in case of recurrences. Higher sensitivity (98%; 95% CI: 91–100%) and specificity (84%; 95% CI: 64–95%) of molecular assays combined with swab sampling, compared to aspirated material, was reported by Adams et al. in a study conducted in Colombia in 2014 [19]. Analogously, Gomes et al. observed that swabs and biopsy specimens have similar sensitivity and accuracy for the diagnosis of American tegumentary leishmaniasis [18]. Moreover, a retrospective study conducted in a referral center in Barcelona in 2021 reported that molecular assays for Leishmania DNA detection were 100% concordant when biopsy and skin swab were performed simultaneously [13]. However, so far, the outcomes of such reports are highly heterogeneous and non-standardized in terms of molecular and sampling techniques, and studies on the matter are lacking especially in the Mediterranean areas [20].

An interesting point regarding the reported case lays in the fact that most studies confirming the good diagnostic performance of the molecular detection of *Leishmania* spp. on superficial skin specimens reported that swab samples were obtained from ulcerated skin lesions [21]. In this case, instead, the skin lesions were dry, with no ulcerated elements or secerning material; however, the performance of a swab sample after scraping the lesion with a blazer in order to uncover the underlying skin tissues was sufficient to demonstrate the presence of *Leishmania* spp. DNA. This allowed us to avoid a second biopsy or the initiation of a specific treatment without a confirmed diagnosis.

An additional aspect worth reflecting upon in this case is the relapse of CL, with new lesions appearing both close to the excision scar and on a different part of the body.

Older age, comorbidities, and lesion duration before treatment are important factors influencing the risk of CL relapse [22]. Immunosuppression also plays a major role in the reactivation of the disease. Many reports in the literature show that anti-TNF agents (such as adalimumab) facilitate secondary dissemination and relapse of CL [23,24], since the amastigotes of *Leishmania* can persist in macrophages for years after the exposure and they can be reactivated in the context of immunosuppression [25].

As for the reactivation of CL in proximity of the biopsy scar, it is important to underline that surgical excision does not guarantee complete eradication of the parasite and is therefore generally not recommended as part of the therapeutic management of CL due to the risk of potential disfiguration, depending on the site of the excision, and relapse [26]. Moreover, the wound healing process after a surgical procedure can impair the immune response in the affected area, resulting in reactivation of CL within a previous surgical scar even when systemic immunosuppression is not documented [27,28].

## 4. Conclusions

Although further studies and standardized protocols are needed, this report supports the relevant diagnostic role of PCR-based swab sampling even on non-ulcerative and non-exudative skin lesions, suggesting that the collection of skin samples through swabs may represent a more patient- and clinician-friendly alternative than biopsy for a first-line PCR diagnosis of CL.

Immunocompromised patients pose additional diagnostic and therapeutic challenges in case of CL, because of the risk of reactivation and the potential involvement of several anatomical regions, therefore requiring a more personalized approach that includes more frequent monitoring and/or immunosuppressive treatment adjustment.

## Data Availability

No new data were created or analyzed in this study. Data sharing is not applicable to this article.
